# Supplementary material for: A Trilayer Dressing with Self-Pumping and pH Monitoring Properties for Promoting Abdominal Wall Defect Repair
Source: Nanomaterials (Basel). 2022 Aug 15;12(16):2802. doi: 10.3390/nano12162802 (PMC9416624; doi:10.3390/nano12162802)
Supplement: Supplementary file 1 [file nanomaterials-12-02802-s001.zip › nanomaterials-1836049-supplementary.pdf]

nanomaterials

Supplementary Material

A Trilayer Dressing with Self-Pumping and pH Monitoring Properties for Promoting Abdominal Wall Defect Repair

Jie Hu [1], Guopu Chen [2] and Gefei Wang [1,2,*]

[1] Department of General Surgery, Jinling Hospital, The First School of Clinical Medicine, Southern Medical University, Guangzhou 510515, China
[2] Research Institute of General Surgery, Jinling Hospital, Medical School of Nanjing University, Nanjing 210002, China
* Correspondence: gefei_w@163.com

Table S1. The primer sequences of mRNA.

Gene	Forward	Reverse
IL-6	CCAGTTGCCTTCTTGGGACT	CTGGTCTGTTGTGGGTGGTA
TNF-α	GAGGCGCTCCCCAAAAAGAT	GCCACGAGCAGGAATGAGAA
IL-10	CTGTGTGAGTAGCAGACCAGT T	TGGCTGGATTGTAGGAGACAGT
IL-4	TCCACGGATGTAACGACAGC	TGGTGTTCCTTGTTGCCGTA
TGF-β	GCTGAACCAAGGAGACGGAA	CCTCGACGTTTGGGACTGAT
PDGF	AACTTCTTGATCTGGCCCCC	ACTTGACGCTGCTGGTGTTA
GAPDH	AGGTTGTCTCCTGTGACTTCAA	CTGTTGCTGTAGCCATATTCATTG
